# Footballer's Lateral Meniscus: Anterior Horn Tears of the Lateral Meniscus with a Stable Knee

**DOI:** 10.5402/2011/170402

**Published:** 2011-05-05

**Authors:** Tetsuo Hagino, Satoshi Ochiai, Eiichi Sato, Yoshiyuki Watanabe, Shinya Senga

**Affiliations:** ^1^The Sports Medicine and Knee Center, Kofu National Hospital, 11-35 Tenjin-cho, Kofu, Yamanashi 400–8533, Japan; ^2^Department of Orthopaedic Surgery, Faculty of Medicine, University of Yamanashi, Yamanashi 409–3898, Japan

## Abstract

This paper aimed to identify the characteristics of isolated anterior horn tear of the lateral meniscus in footballers who underwent arthroscopic surgery. We identified 8 patients with stable knee and no ligament injury, who had only isolated anterior horn tear of the lateral meniscus between 2007 and 2009. All 8 patients were footballers, comprising 7 men and 1 woman with mean age of 18.6 years. Arthroscopy revealed multiple longitudinal tears in 2 patients, longitudinal tear in 2 patients, degenerative tear in 3 patients, and flap tear in 1 patient. Two patients were treated by repair, five by partial excision, and one by rasping only. The mean Lysholm score was 65 before surgery and recovered to 89 at the last followup, on average 12 months after surgery. Anterior horn tear of the lateral meniscus in footballers with a stable knee is characterized by pain at the anterolateral aspect of the knee during knee extension, especially when kicking a ball, and pain during weight-bearing knee extension, together with MRI finding of hyperintense signal in the anterior horn of the lateral meniscus. Preoperative diagnosis may be possible based on these findings in footballers.

## 1. Introduction

Meniscus tear is a common knee injury. The prevalence is high from the late teens to the twenties and is a frequent cause of temporary cessation of sport activities. In playing football, a sudden change in direction may result in meniscus tear. Meniscus tear occurs in approximately 50% of the active football athletes, and the frequency increases when a football athlete has 6–10 years of experience [1]. Most of the injuries are found in the posterior horn of the meniscus, and an isolated tear in the anterior horn is rare [2]. In the present study, all the patients who had an isolated anterior horn tear of the lateral meniscus and underwent arthroscopic surgery were footballers, suggesting that there are common features associated with this injury. We, therefore, investigated the symptoms, MRI findings, arthroscopic findings, and other clinical findings of these cases. The objective of this study was to identify the characteristics of anterior horn tear of the lateral meniscus in footballers.

## 2. Patients and Methods

Among 885 patients who underwent arthroscopic surgeries in our center between January 2007 and December 2009, 499 patients had meniscus tears, 10 of whom had tears only in the anterior horn of the lateral meniscus (zone D [3]). Of these 10 patients, 8 with stable knees and no ligament injury were studied. Patients who had any concomitant lesion of the midbody or posterior horn of the lateral meniscus, or any lesion of the medial meniscus, were excluded from this study. The characteristics of these 8 patients, including physical findings, MRI findings, and arthroscopic findings were investigated retrospectively.

## 3. Results

All 8 patients were footballers; 7 were a soccer players and 1 was futsal player. Five were senior high school students, 2 were university students, and 1 belonged to a club team. The mean duration of playing in competitions was 9.3 (range: 7 to 12) years. There were 7 men and 1 woman with a mean age of 18.6 (range: 16 to 22) years. The injury was in the right knee in 5 patients and in the left knee in 3 patients. The mean interval from injury to surgery was 6.9 (range: 1 to 21) months. The pain first started while kicking a ball in 4 patients and during contact with other players in 2 patients, while the situation of injury was unclear in 2 patients. Of 8 patients, 1 complained of catching and 2 of a sense of giving away. 

**Figure 1:**
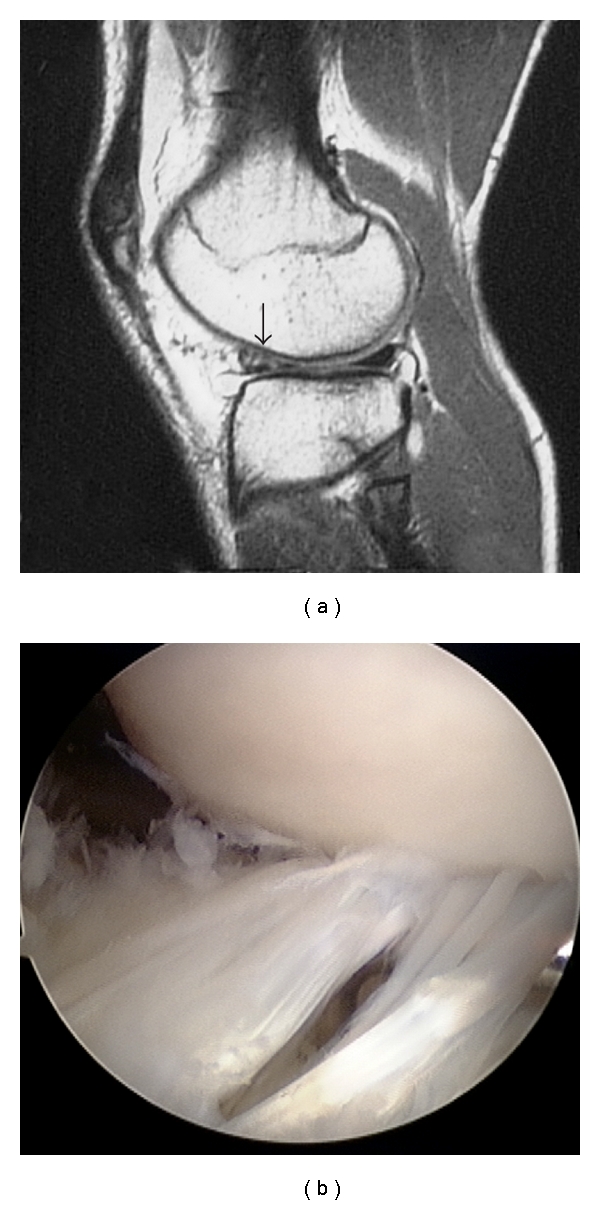
A 17-year-old male senior high school soccer player. (a) Sagittal T2-weighted MRI shows hyperintense signal (arrow) in the anterior horn of the lateral meniscus. (b) Arthroscopic image shows multiple longitudinal tears in the anterior horn of the lateral meniscus.

**Figure 2:**
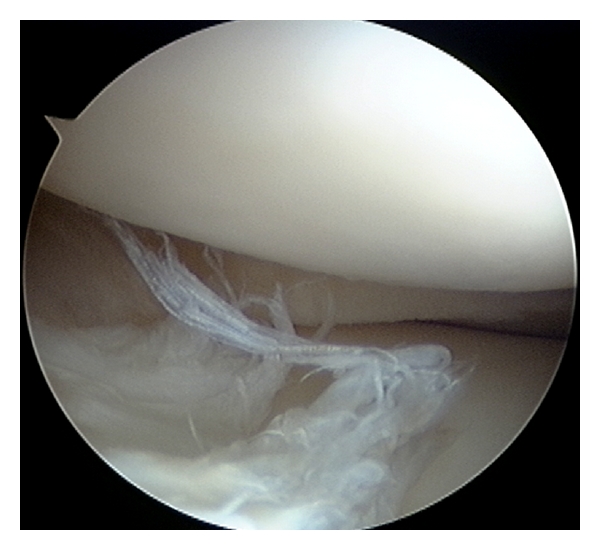
Arthroscopic finding of a 22-year-old male futsal player. Degenerative tear is observed in the anterior horn of the lateral meniscus.

**Figure 3:**
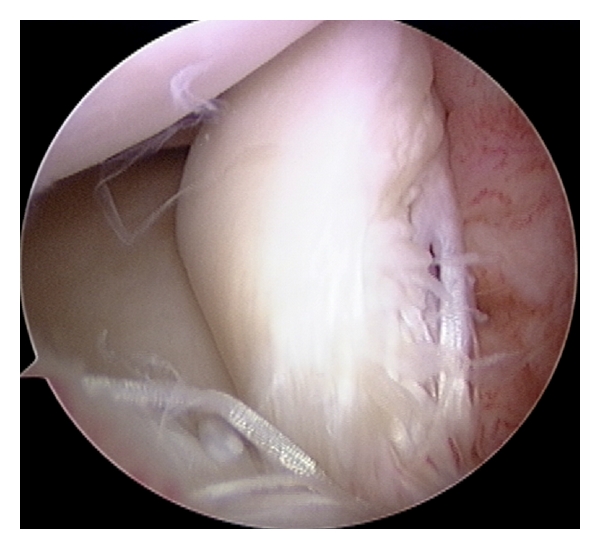
Arthroscopic finding of a 21-year-old male university soccer player. A flap tear is observed.

At the initial examination, all patients reported pain at the anterolateral aspect of the knee joint during load-bearing knee extension, and also pain while kicking a ball. Two patients had effusion, 6 had tenderness at the lateral joint line, and 3 had a positive McMurray test. Magnetic resonance imaging (MRI) examination showed a hyperintense signal in the anterior horn of the lateral meniscus on a T2-weighted sagittal image in all patients (Figure 1(a)). Two demonstrated a linear hyperintense signal, 5 showed multiple hyperintense signals suggesting degenerative change, and 1 had irregular meniscal surface. Arthroscopy revealed multiple longitudinal tears in 2 patients (Figure 1(b)), longitudinal tear in 2 patients, degenerative tear in 3 patients (Figure 2), and flap tear in 1 patient (Figure 3). 

Two patients were treated by repair, 5 patients by partial excision, and 1 patient by rasping only. Repair was performed by suturing using the meniscal Viper repair system. The mean Lysholm score was 65 (range: 62 to 69) before surgery and recovered to 89 (range: 73 to 100) at the last followup, on average 12 (range: 6 to 20) months after surgery. All patients returned to their previous levels of football.

## 4. Discussion

In their 10-year study on the epidemiology of athletic knee injuries, Majewski et al. [4] reported that among a total of 7769 knee injuries, 836 (10.8%) were medial meniscus tears while 284 (3.66%) were lateral meniscus tears, with a ratio of 3 to 1. Terzidis et al. [5] reported that meniscal tears commonly occur in combination with anterior cruciate ligament tears or other intra-articular lesions, and that isolated meniscal tears are rare. In their report, most of the tears were a result of football, basketball or ski injuries, comprising 69.3% medial meniscus tears and 30.7% lateral meniscus tears. Furthermore, among 116 cases of lateral meniscus tear, there were only 3 cases (2.5%) of isolated anterior horn tear. In the present series, isolated anterior horn (zone D) tear was even rarer; it was found in only 8 cases (1.6%) among a total of 499 cases of meniscus tear. 

All 8 patients were soccer or futsal players. All reported pain at the anterolateral aspect of the knee joint during knee extension and pain appearing while kicking a ball, suggesting a relation between the ball kicking movement and meniscus tear. Furthermore, degenerative or multiple longitudinal tear was observed in 5 patients, suggesting that the tear may be caused by repeated kicking of the ball. Choi and Victoroff**** [6] investigated the anterior horn tear of the lateral meniscus in 14 soccer players and concluded that the common arthroscopic findings included multiple longitudinal tears within the white zone of the anterior horn. They considered McMurray test and joint-line tenderness not useful for the diagnosis. On the other hand, Eren [7] investigated the usefulness of joint-line tenderness by physical examination in the diagnosis of meniscal tears, and concluded that joint-line tenderness as a test for lateral meniscal tears is accurate (96%), sensitive (89%), and specific (97%). In the present study, the McMurray test was positive in 3 of 8 cases and joint-line tenderness was observed in 6 of 8 cases. These results indicate that these tests may contribute to the diagnosis. 

The characteristic physical finding of anterior horn tear is pain at the anterolateral aspect of the knee during knee extension, especially when kicking a ball, and pain during weight-bearing extension. These symptoms were observed in all 8 patients. Although MRI has been reported to have a low diagnostic rate for anterior horn tear of the lateral meniscus because of the high false-positive rate [8], Choi and Victoroff [6] showed that MRI was useful for the diagnosis. Furthermore, De Smet and Mukherjee [9] reported a high sensitivity of MRI for the diagnosis of anterior horn tear of the lateral meniscus. In the present study, T2-weighted MRI demonstrated a hyperintense signal in the anterior horn of the lateral meniscus in all 8 patients, supporting the usefulness of MRI examination. This study indicates that a preoperative diagnosis of anterior horn tear of the lateral meniscus may be possible based on the characteristic MRI together with physical findings in a footballer.

## References

[1] Osteaux M, Meirleir KD, Shahabpour M (1991). *Resonance Imaging and Spectroscopy in Sports Medicine*.

[2] Shepard MF, Hunter DM, Davies MR, Shapiro MS, Seeger LL (2002). The clinical significance of anterior horn meniscal tears diagnosed on magnetic resonance images. *American Journal of Sports Medicine*.

[3] Cooper DE, Arnoczky SP, Warren RF (1990). Arthroscopic meniscal repair. *Clinics in Sports Medicine*.

[4] Majewski M, Susanne H, Klaus S (2006). Epidemiology of athletic knee injuries: a 10-year study. *Knee*.

[5] Terzidis IP, Christodoulou A, Ploumis A, Givissis P, Natsis K, Koimtzis M (2006). Meniscal tear characteristics in young athletes with a stable knee: arthroscopic evaluation. *American Journal of Sports Medicine*.

[6] Choi NH, Victoroff BN (2006). Anterior horn tears of the lateral meniscus in soccer players. *Arthroscopy*.

[7] Eren OT (2003). The accuracy of joint line tenderness by physical examination in the diagnosis of meniscal tears. *Arthroscopy*.

[8] Shankman S, Beltran J, Melamed E, Rosenberg ZS (1997). Anterior horn of the lateral meniscus: another potential pitfall in MR imaging of the knee. *Radiology*.

[9] De Smet AA, Mukherjee R (2008). Clinical, MRI, and arthroscopic findings associated with failure to diagnose a lateral meniscal tear on knee MRI. *American Journal of Roentgenology*.

